# Editorial: The non-coding RNA world in animals and plants

**DOI:** 10.3389/fgene.2025.1558406

**Published:** 2025-02-07

**Authors:** Lingling Wang, Bin Hu, Min-Jin Han, Q.-Z. Zhou

**Affiliations:** ^1^ Ministry of Education Key Laboratory for Ecology of Tropical Islands, Key Laboratory of Tropical Animal and Plant Ecology of Hainan Province, College of Life Sciences, Hainan Normal University, Haikou, China; ^2^ Rubber Research Institute, Chinese Academy of Tropical Agricultural Sciences, Haikou, China; ^3^ State Key Laboratory of Resource Insects, Institute of Sericulture and Systems Biology, Southwest University, Chongqing, China; ^4^ Key Laboratory of Sericultural Biology and Genetic Breeding, Ministry of Agriculture and Rural Affairs, College of Sericulture, Textile and Biomass Sciences, Southwest University, Chongqing, China; ^5^ DUKE-NUS Medical School, National University of Singapore, Singapore, Singapore

**Keywords:** non-coding RNAs, long noncoding RNA, microRNA, plant, animal

Non-coding RNAs (ncRNAs) are transcriptionally produced and do not code proteins, with a spectrum of diversified type and function (Chen and Kim, 2024). Although ncRNAs have many types, including the long noncoding RNAs (lncRNAs), microRNAs (miRNAs), small nucleolar RNAs (snoRNAs), transfer RNA (tRNA), ribosomal RNA (rRNA), and circular RNA (circRNA), most of them are lncRNAs and miRNAs. In 1984, the first regulatory ncRNA *microRNA F* (*micF*) was discovered from *Escherichia coli*. *MicF* inhibits the translation of OmpF by binding to its ribosome-binding site, blocking ribosomal entry (Mizuno et al., 1984; Corcoran et al., 2012).

Over the past 40 years, numerous regulatory ncRNAs, such as *Let-7*, *enod40*, *H19*, and *NEAT1*, have been identified as key regulators in animals or plants (Bartolomei et al., 1991; Crespi et al., 1994; Pasquinelli et al., 2000; Hutchinson et al., 2007). Studies have demonstrated the crucial roles of ncRNAs—particularly miRNAs and lncRNAs—in development, metabolism, and disease (Kallen et al., 2013; Tripathi et al., 2013; Chen and Kim, 2024). However, most of the ncRNA world is still unknown.

To provide new insights and a deeper understanding of the functional and regulatory roles of ncRNAs, we curated six articles for the “*The Non-Coding RNA World in Animals and Plants*” topic. These papers include original research on ncRNAs in tobacco, common carp (*Cyprinus carpio* L.), and pulmonary hypertension, as well as reviews on ncRNAs in endocrine disorders, eye diseases, and intrahepatic cholestasis of pregnancy (ICP).

Xie et al. analyzed 549 publicly available RNA-seq datasets on tobacco and identified 30,212 lncRNAs (Xie et al.). These lncRNAs exhibit distinct characteristics compared to coding genes, including fewer exons, higher A/U content, and greater tissue specificity. Functional analysis of the potential targets of these lncRNAs revealed their association with nicotine biosynthesis; their findings further validated through topping treatment. This study advances our understanding of the functional roles of lncRNAs in tobacco and provides new candidate genes for regulating nicotine production.

Ledesma–Pacheco et al. presented our current understanding regarding miRNAs’ regulatory mechanism of endocrine disorders and their potential influence as disease biomarkers during their development processes by overviewing recent and significant research outputs (Ledesma-Pacheco et al.). This review illuminated the most recent information related to the potential functions of miRNAs in endocrine disorders, including diabetes mellitus, thyroid diseases, and osteoporosis, and their novel diagnostic and therapeutic purposes. It will enhance our knowledge of miRNAs’ roles in endocrine disorders and facilitate the development of novel miRNA-based diagnostic and therapeutic tools for studying endocrine disorders.

Benavides-Aguilar et al. integrated recent studies on miRNAs in common eye diseases to illustrate the regulatory roles of miRNAs in the eye-related diseases such as cataracts, glaucoma, and macular degeneration (Benavides-Aguilar et al.). This review provides valuable insights into the potential applications of miRNAs in the prognosis and treatment of these eye-related disorders.

Das et al. integrated 468 raw RNA-seq datasets from 28 tissues in common carp (*C. carpio* L.), a substitute vertebrate fish model for zebrafish, to identify lncRNAs and circular RNAs using various bioinformatics tools (Das et al.). They conducted the lncRNA–miRNA–mRNA interaction network analysis and introduced CCncRNAdb, a comprehensive web resource (http://backlin.cabgrid.res.in/ccncrnadb/) to facilitate further exploration of ncRNAs in common carp.

Xiong et al. consolidated recent discoveries on miRNAs, lncRNAs, circRNAs, etc. for ICP to discuss their potential as diagnostic markers, prognostic tools, and therapeutic targets, offering a foundation for improving early detection and personalized treatment of ICP (Xiong et al.).

Chen et al. conducted a comprehensive study on the peripheral blood samples of patients with pulmonary hypertension (PH) and healthy individuals through whole-genome miRNA sequencing and transcriptome analysis, exploring the potential role of miRNAs in PH (Chen et al.). By screening the differentially expressed miRNAs, they identified four miRNAs (hsa-miR-1304-3p, hsa-miR-490-3p, hsa-miR-11400, and hsa-miR-31-5p) as potential clinical diagnostic biomarkers for PH. This finding provides a valuable foundation for further understanding the specific role of miRNAs in the mechanisms of PH, opening new avenues for early diagnosis and precision medicine in PH.

In summary, these articles expand our current knowledge of ncRNAs and deepen our understanding of the non-coding RNA world.
